# Compositional Analysis of Extracellular Aggregates in the Eyes of Patients With Exfoliation Syndrome and Exfoliation Glaucoma

**DOI:** 10.1167/iovs.62.15.27

**Published:** 2021-12-29

**Authors:** Alicia De Maria, Keith D. Zientek, Larry L. David, Phillip A. Wilmarth, Anjali M. Bhorade, George J. Harocopos, Andrew J W. Huang, Augustine R. Hong, Carla J. Siegfried, Linda M. Tsai, Arsham Sheybani, Steven Bassnett

**Affiliations:** 1 Department of Ophthalmology & Visual Sciences, Washington University School of Medicine, St. Louis, Missouri, United States; 2 Proteomics Shared Resource, Oregon Health and Science University, Portland, Oregon, United States; 3 Department of Chemical Physiology & Biochemistry, Oregon Health and Science University, Portland, Oregon, United States; 4 Department of Pathology & Immunology, Washington University School of Medicine, St. Louis, Missouri, United States

**Keywords:** exfoliation, proteome, LEFTY2, glaucoma, syndrome

## Abstract

**Purpose:**

Exfoliation syndrome (XFS) is a condition characterized by the production of insoluble fibrillar aggregates (exfoliation material; XFM) in the eye and elsewhere. Many patients with XFS progress to exfoliation glaucoma (XFG), a significant cause of global blindness. We used quantitative mass spectrometry to analyze the composition of XFM in lens capsule specimens and in aqueous humor (AH) samples from patients with XFS, patients with XFG and unaffected individuals.

**Methods:**

Pieces of lens capsule and samples of AH were obtained with consent from patients undergoing cataract surgery. Tryptic digests of capsule or AH were analyzed by high-performance liquid chromatography–mass spectrometry and relative differences between samples were quantified using the tandem mass tag technique. The distribution of XFM on the capsular surface was visualized by SEM and super-resolution light microscopy.

**Results:**

A small set of proteins was consistently upregulated in capsule samples from patients with XFS and patients with XFG, including microfibril components fibrillin-1, latent transforming growth factor-β–binding protein-2 and latent transforming growth factor-β–binding protein-3. Lysyl oxidase-like 1, a cross-linking enzyme associated with XFS in genetic studies, was an abundant XFM constituent. Ligands of the transforming growth factor-β superfamily were prominent, including LEFTY2, a protein best known for its role in establishing the embryonic body axis. Elevated levels of LEFTY2 were also detected in AH from patients with XFG, a finding confirmed subsequently by ELISA.

**Conclusions:**

This analysis verified the presence of suspected XFM proteins and identified novel components. Quantitative comparisons between patient samples revealed a consistent XFM proteome characterized by strong expression of fibrillin-1, lysyl oxidase-like-1, and LEFTY2. Elevated levels of LEFTY2 in the AH of patients with XFG may serve as a biomarker for the disease.

Exfoliation syndrome (XFS; MIM#177650) is an age-related disorder characterized by the deposition of fibrillar aggregates (exfoliation material [XFM]) in extracellular matrices throughout the body.^1^ Meta-analysis suggests an association between the presence of XFM and a modestly increased risk of cardiovascular and cerebrovascular disease.^2^ It is in the eye, however, where the accumulation of XFM seems to be most consequential, with almost half of patients with XFS eventually developing exfoliation glaucoma (XFG), a relatively intractable form of the disease that often responds poorly to medical therapies.^3^^,^^4^ Patients with XFS are also prone to other ocular conditions, including cataract,^5^ lens subluxation (after rupture of the ciliary zonule),^6^ decreased corneal endothelial cell density,^7^ keratopathy, and iris transillumination defects.^8^

Under slit lamp examination, XFM can be detected throughout the anterior segment of affected individuals. It is especially conspicuous at the pupillary margin and on the anterior lens capsule, where it accumulates as a grey, dusty layer studded with larger (2–10 µm diameter) aggregates. As noted in 1917 by Lindberg in his initial description, XFM is often distributed in a series of concentric zones on the lens surface.^9^ A faint central disk (CD), corresponding approximately to the diameter of the pupil, is visible in about one-third of patients.^10^^,^^11^ Nearer the lens equator is the granular zone (GZ); its frosted appearance signifying the presence of densely packed XFM aggregates. Between the CD and the GZ, the clear zone (CZ) is swept free of XFM deposits, presumably by the passage of the iris during constriction and dilation of the pupil.^12^

XFM is composed of rough-surfaced fibers, 15 to 40 nm in thickness, with cores consisting of two or more microfibrils twisted together.^13^ Because the presence of XFM is linked strongly to glaucoma risk, studies have sought to determine its molecular composition, using immunocytochemical^14^^,^^15^ or mass spectrometric approaches.^16^^–^^18^ Capsulorhexis specimens (circular flaps of anterior lens capsule removed in the course of cataract surgery) have been widely used for such studies. The pieces of capsule are about 5 mm in diameter, with a layer of adherent lens epithelial cells on one side and, in the case of patients with XFS, deposits of XFM on the other. Over the last two decades, a tentative proteome for XFM has emerged, with more than fifty putative constituents,^18^^,^^19^ most belonging to the set of extracellular matrix–associated proteins known as the matrisome.^20^ Members of the elastic fiber system (such as fibrillin, elastin, and latent transforming growth factor β [TGFβ]-binding proteins [LTBPs]) have been detected consistently in XFM, along with basal lamina proteins (such as fibronectin and laminin), complement components, clusterin, and lens crystallin proteins.^19^

Uncertainties remain, however, about the precise composition of the XFM, in part because the material is highly cross-linked and therefore difficult to analyze. There are also technical challenges in removing the thin layer of XFM from the capsule surface and it has often been necessary to combine samples to have sufficient material for analysis. Consequently, it is unclear whether XFM composition differs quantitatively or qualitatively between patients. Studies have also focused on those proteins that accumulate in the eyes of patients with XFS, neglecting the possibility that some proteins might be depleted.

For our analysis, we used tandem mass tagging (TMT), a quantitative mass spectrometric approach, to compare the composition of capsule specimens and AH samples from control patients, patients with XFS, and patients with XFG.^21^ The results provided further insights into the composition of this complex material.

## Methods

### Patients

The Institutional Review Board of Washington University School of Medicine approved this study (IRB # 201802119) in compliance with the tenets of the Declaration of Helsinki and HIPAA guidelines. Samples were obtained from patients undergoing cataract surgery at the Washington University Ophthalmology Service. Eight ophthalmic surgeons collected the samples used in the study. Patients with XFS were diagnosed based on the presence of visible particulate material on the pupillary ruff, the corneal endothelium, or the lens surface. Patients with XFS and glaucomatous cupping, retinal nerve fiber thinning (as measured by optical coherence tomography), and/or visual field loss, were categorized as patients with XFG. Control samples were obtained from patients with cataract and other forms of glaucoma (CAT/GL) or patients with cataract but no ocular comorbidities (CAT). Most of the CAT/GL patients were diagnosed with primary open-angle glaucoma, although other types (e.g., pigmentary glaucoma) were also included in the study. Patient clinical and demographic data are shown in Supplementary Table S1.

After obtaining informed consent, patients were transferred to the operating room, where standard sterile technique was used to prepare the eye for surgery. A 1.0 mm paracentesis was created and a sample of AH (≈0.1 mL) was withdrawn using a 27G blunt cannula attached to a 1-mL syringe. The AH was placed in a microfuge tube and immediately frozen on dry ice. The anterior chamber was reformed with balanced salt solution, followed by injection of an ophthalmic viscoelastic. Next, a temporal clear-corneal or scleral-tunnel incision was created, through which capsulorhexis forceps were introduced and used to complete a continuous curvilinear capsulorhexis. The resulting flap of lens capsule with an attached layer of epithelial cells was removed and placed in a microfuge tube containing sterile tissue culture medium. Laboratory staff were given prior notice of the surgery schedule and were on hand to pick up the capsule specimens immediately after the capsulorhexis was performed. Samples were transported directly to the laboratory (located in an adjacent building). Capsule samples were decellularized by immersion in 0.5% sodium deoxycholate in water. The decellularized lens capsules and the AH samples were frozen at −80 °C until used for mass spectrometric analysis. All samples (XFS, XFG, CAT, and CAT/GL) were handled identically.

### SEM

Lens capsule specimens from control patients, patients with XFS, or patients with XFG were fixed in 2% paraformaldehyde/2.5% glutaraldehyde (Electron Microscopy Sciences, Hatfield, PA) in PBS (pH 7.4) for at least 3 days. After fixation, samples were rinsed with 0.15 M sodium cacodylate buffer (pH 7.4) containing 2 mM CaCl_2_, followed by secondary fixation in 1% OsO_4_. Samples were dehydrated in a graded ethanol series and critical point dried (Leica EM CPD 300, Vienna, Austria). Capsule samples were mounted on specimen holders, sputter-coated with iridium (Leica ACE600), and examined using a Zeiss Merlin FE-SEM microscope operating at 2 kV.

### Immunofluorescence

Capsule specimens were fixed overnight at 4 °C in 4% paraformaldehyde/PBS. Samples were then incubated in 0.5% triton X-100/PBS for 15 minutes and placed in blocking solution (5% BSA/PBS) for 2 hours at room temperature. Primary antibodies (diluted in blocking solution) were applied overnight at 4 °C. Samples were then washed in PBS and incubated for 2 hours with appropriate secondary antibodies. Hoechst 33342 (NucBlue, Thermofisher) was used as a nuclear counterstain. Samples were mounted with Vectashield (Vectorlabs), coverslipped, and imaged using Airyscan mode on a Zeiss LSM800 confocal microscope. All immunofluorescence experiments were performed on at least three independent samples from each category (XFS, XFG, etc.). The primary antibodies used in this study are shown in Table 1.

**Table 1.** Primary Antibodies Used in Immunofluorescence Studies of XFM on Lens Capsule Samples

Protein	Clonality	Dilution	Catalog #	Company
FBN1	Monoclonal (11C1.3)	1:40	GTX23090	GeneTex, Irvine, CA
Microfibril associated protein-2 (MFAP2)	polyclonal	1:100	PA5-52425	Thermofisher Scientific, Waltham, MA
Latent TGFβ-binding protein-2 (LTBP2)	Polyclonal	1:100	NBP1-88411	Novus Biologicals, Littleton, CO
Lysyl oxidase-like 1 (LOXL1) pro-enzyme	Polyclonal	1:100	H00004016-D01P	Abnova, Taipei, Taiwan
LOXL1 pro-domain	Monoclonal (H-11)	1:100	Sc-166632	Santa Cruz Biotechnology
LOXL1 catalytic domain	Polyclonal	1:100	PB9719	Boster, Pleasanton, CA

### Left–Right Determination Factor 2 (LEFTY2) ELISA

The concentration of LEFTY2 in AH samples was measured using a commercial sandwich ELISA kit (Cat# OKCA01327; Aviva systems biology, San Diego, CA), according to the manufacturer's instructions. In brief, 0.1 mL of AH were used for the assay, which was performed in a 96-well plate format. Recombinant human LEFTY2 was used to generate standard curves. The levels of bound biotinylated detector antibody were quantified by avidin-peroxidase activity with 3,3′,5,5′-tetramethylbenzidine as a substrate and absorbance was measured at 450 nm in a plate reader (Spectramax 190, Molecular Devices, San Jose, CA).

### Trypsinization of Capsule and AH Samples

Capsules stored in 1.5 mL polypropylene centrifuge tubes were thawed and 150 µL of 5% SDS, 50 mM triethylammonium bicarbonate (TEAB) were added. Samples were vortexed for 30 seconds and homogenized at 4 °C using a Bioruptor Pico sonication device (Diagenode Diagnostics, Seraing, Belgium, Cat # B01060010) by adding 30 mg of extraction beads (Cat # C20000021) and sonicating for 20 cycles of 30 seconds on/30 seconds off. Sonication was performed without transfer to special Diagenode tubes to avoid sample loss. A Pierce BCA protein assay (ThermoFisher Scientific, Waltham, MA) was then performed. Samples contained 21 to 55 µg of protein (average = 39 µg). One-half (75 µL) of each sample was transferred to 1.5 mL Lobind tubes (Eppendorf, Enfield, CT), 3.4 µL of 0.5 M dithiothreitol were added, and samples were incubated at 95 °C for 10 minutes. After this reduction step, samples were alkylated by addition of 7.8 µL of 0.5 M iodoacetamide and incubation at room temperature for 30 minutes in the dark. Samples were then acidified by addition of 8.5 µL of 12% phosphoric acid and 562 µL of 90% methanol, 100 mM TEAB. Samples were then transferred ¼ at a time to S-trap micro columns (Protifi, Farmingdale, NY) inserted into 1.5-mL centrifuge tubes, and the liquid was passed through following each addition by centrifugation at 4000×*g* for 3 minutes. S-traps were then washed six times by adding 150 µL 90% methanol, 100 mM TEAB and centrifuging as described elsewhere in this article, rotating the S-traps 180° between each wash. S-traps were then transferred to 1.5 mL Lobind centrifuge tubes, 40 µL of 80 ng/µL sequencing grade modified trypsin (Promega, Fitchburg, WI, Cat # V5111) in 50 mM TEAB were added. The samples were loosely capped and digestion was performed at 37 °C overnight in a humidified chamber. Upon completion of digestion, 20 µL of 50 mM TEAB were added, the samples were incubated at 37 °C for 15 minutes, and peptides were eluted by sequential addition of 50 µL of TEAB, 50 µL 0.2% aqueous formic acid, and 50 µL of 50% acetonitrile, with centrifugation at 4000×*g* for 4 minutes. The combined fractions were dried by vacuum centrifugation, 100 µL of 50% methanol were added, and the samples were again dried. Samples were dissolved by vigorous shaking in 100 µL of water and peptide concentrations were determined using a Pierce Quantitative Colorimetric Peptide Assay (ThermoFisher Scientific, Cat # 23275). Approximately 7.5 µg of peptide from each capsule sample were dried by vacuum centrifugation and redissolved by vigorous shaking in 20 µL of 100 mM TEAB.

Preparation of AH digests was similar, except 75 µl of each thawed AH sample had 19 µL of 20% SDS added and samples simply vortexed instead of performing bead sonication. A Pierce BCA protein assay was performed, and from 107 to 373 µg of protein were obtained (average = 234 µg). Each AH sample (75 µL) was then digested using the S-trap protocol, as described above for the capsule samples. Following resolubilization of the peptide digest in 100 µL of water, a peptide assay was performed as above, and 10 µg of peptide from each AH sample dried and dissolved in 20 µL of 100 mM TEAB.

### TMT Labeling and Normalization Run

For labeling of capsule digests, 200 µg portions of TMT 10-plex reagents (ThermoFisher Scientific, Cat # 90309) and TMT11-131C reagent (Cat # PIA34807) were dissolved in 12 µL of anhydrous acetonitrile (ACN). Peptide samples (20 µL) were immediately mixed with these reagents and labeling was performed by shaking at room temp for 1 hour. For labeling of AH digests, 16-plex TMPpro reagents in 500 µg portions (Cat # A44522) were used, by dissolving each in 52 µL of ACN, and mixing 12 µL (116 µg reagent) with 20 µL of digest containing 10 µg of peptide. Following labeling, 2 µL of each labeled peptide from either capsule or AH digests were pooled, 2 µL of 5% hydroxylamine were added, samples were incubated at room temp for 15 minutes and then dried by vacuum centrifugation. The remaining 30 µL of each labeled sample were frozen at −70 °C without hydroxylamine addition, in case relabeling was required. The pooled samples were dissolved in 20 µL of 5% formic acid and 2 µg of peptides were analyzed by a single 2 hour liquid chromatography tandem mass spectrometry (LC-MS/MS) method using an Orbitrap Fusion instrument, as described elsewhere in this article. These runs were performed to check labeling efficiency (>90%) and to volumetrically normalize each sample to provide similar total reporter ion intensities for each labeled sample in the pool before the two-dimensional liquid chromatography/mass spectrometry analysis, using the informatics pipeline described elsewhere in this article. The remaining 30 µL portion of each TMT-labeled sample was then thawed, 2 µL of 5% hydroxylamine were added, and samples were incubated at room temp for 15 minutes. Peptides (19 µg in the first experiment, 44 µg in the second experiment, and 55 µg in the AH analysis) were dried in preparation for two-dimensional liquid chromatography/mass spectrometry analysis.

### Two-Dimensional Liquid Chromatography/Mass Spectrometry (2D-LC/MS) Analysis

The multiplexed capsule samples and the AH samples were dissolved in 10 mM ammonium formate (pH 9) and injected onto a NanoEase 5 µm XBridge BEH130 C18 300 µm x 50 mm column (Waters Corporation, Milford, MA) at 3 µL/min in a mobile phase containing 10 mM ammonium formate (pH 9). Peptides from the capsules were eluted by sequential injection of 20 µL volumes of either 14%, 20%, 25%, 30%, 50%, and 90% ACN (6 fractions – first experiment) or 17%, 21%, 23%, 25%, 27%, 29%, 31%, 33%, 35%, 40%, and 90% ACN (11 fractions – second experiment) in 10 mM ammonium formate (pH 9) at 3 µL/min flow rate, while peptides from AH were eluted using 17%, 20%, 21%, 22%, 23%, 24%, 25%, 26%, 27%, 28%, 29%, 30%, 32%, 35%, 37%, 40%, 50%, and 90% ACN (18 fractions). Eluted peptides were diluted at a three-way union with a mobile phase containing 0.1% formic acid at 24 µL/min flow rate and delivered to an Acclaim PepMap 100 µm x 2 cm NanoViper C18, 5 µm trap (Thermo Fisher Scientific) on a switching valve.

After 10 minutes of loading, the trap column was switched on-line to a PepMap RSLC C18, 2 µm, 75 µm × 25 cm EasySpray column (ThermoFisher Scientific). TMT 11-plex labeled peptides from capsules were then separated at low pH in the second dimension using a 7.5% to 30% ACN gradient over 83 minutes in mobile phase containing 0.1% formic acid at 300 nL/min flow rate, while TMT 16-plex labeled peptides from AH were separated using a 5% to 25% ACN gradient over 100 minutes. Tandem mass spectrometry data were collected using an Orbitrap Fusion Tribrid instrument (Thermo Scientific, San Jose, CA) configured with an EasySpray NanoSource. Survey scans were performed in the Orbitrap mass analyzer (resolution = 120,000), with internal mass calibration enabled, and data-dependent MS2 scans using dynamic exclusion performed in the linear ion trap using collision-induced dissociation. Reporter ion detection was performed in the Orbitrap mass analyzer using MS3 scans after synchronous precursor isolation of the top 10 ions in the linear ion trap, and higher energy collisional dissociation in the ion-routing multipole. Instrument parameters for the three individual TMT experiments are provided in Supplementary Table S2.

### Bioinformatics Analysis Pipeline

The binary instrument files were processed with the PAW pipeline.^22^ Binary files were converted to text files using MSConvert.^23^ Python scripts extracted TMT reporter ion peak heights and fragment ion spectra in MS2 format.^24^ The Comet search engine (version 2016.03)^25^ was used: 1.25 Da monoisotopic peptide mass tolerance, 1.0005 Da monoisotopic fragment ion tolerance, fully tryptic cleavage with up to two missed cleavages, variable oxidation of methionine residues, static alkylation of cysteines, and static modifications for TMT labels (at peptide *N*-termini and at lysine residues). Searches used UniProt proteome UP000005640 (human, taxon ID 9606) canonical FASTA sequences (20,603 proteins). Common contaminants (174 sequences excluding any albumins) were added, and sequence-reversed entries were concatenated for a final protein FASTA file of 41,554 sequences.

Top-scoring peptide spectrum matches were filtered to a 1% false discovery rate using interactive delta-mass and conditional PeptideProphet-like linear discriminant function scores.^26^ Incorrect delta-mass and score histogram distributions were estimated using the target/decoy method.^27^ The filtered peptide spectrum matches were assembled into protein lists using basic and extended parsimony principles and required two distinct peptides per protein. The final list of identified proteins, protein groups, and protein families were used to define unique and shared peptides for quantitative use. Total (summed) reporter ion intensities were computed from the peptide spectrum matches associated with all unique peptides for each protein.

The protein intensity values for each biological sample in each biological condition were compared for differential protein expression using the Bioconductor package edgeR^28^ within Jupyter notebooks. Result tables contained typical proteomics summaries, reporter ion intensities, and statistical testing results.

## Results

Morphologic or biochemical analyses were performed on surgical specimens from patients who underwent cataract surgery over a fifteen-month period, from November 2018 to February 2020. Patients were divided into four groups (Supplementary Table S1). The first group (CAT) consisted of patients with age-related cataract but no ocular comorbidities. The second group (CAT/GL) consisted of patients with cataract and glaucoma. The third group (XFS) consisted of patients with cataract and XFS. The fourth group (XFG) comprised patients with cataract and XFG. For each patient, a circular flap of anterior lens capsule, a sample of AH, or both, were collected and analyzed.

### Morphologic Analysis

**Figure 1.**
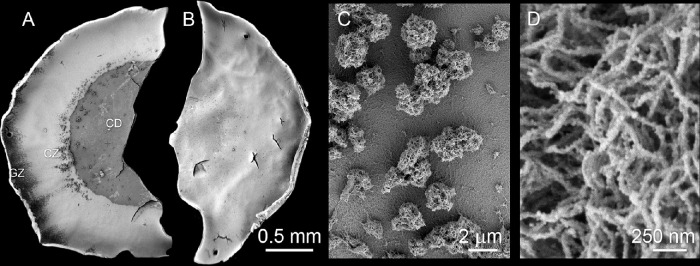
Distribution of XFM on the lens capsule surface as visualized by SEM. (**A**) Hemisected capsulorhexis specimen with XFM (*darker material*) distributed in three regions: central disk (CD), clear zone (CZ) and granular zone (GZ). (**B**) Control sample (CAT/GL) from a glaucoma patient without XFS. Note the absence of XFM deposits from the capsular surface. (**C**) Higher magnification view of XFM aggregates from the CD region of an XFS lens. (**D**) The XFM is composed of tangled fibrils approximately 30 nm in diameter with a roughly textured surface.

In most capsule specimens from patients with XFS or patients with XFG, a CD, CZ, and a variable portion (depending on the diameter of the capsulorhexis) of the GZ (see Fig. 1A), were evident by SEM. The XFM was present as a thin layer in the CD, as densely packed aggregates in the GZ, and as sparse aggregates strewn along the border between the CD and the CZ. By contrast, the capsular surfaces of control samples (CAT or CAT/GL) were generally smooth and featureless (Fig. 1B). XFM aggregates were 2 to 20 µm in diameter (Figs. 1A and C), composed of tangled, rough-surfaced fibrils approximately 30 nm thick (Fig. 1D). Structurally indistinguishable fibers were observed in all samples from affected individuals (four XFS and three XFG lens capsules) examined by SEM (data not shown), although some samples were more heavily laden with XFM than others. Similarly, samples from CAT (*n* = 10) and CAT/GL patients (*n* = 12) were indistinguishable from each other, and all were similar in appearance to the example shown in Figure 1B. Thus, the distribution of XFM across the lens surface and the ultrastructural appearance of individual XFM fibers were consistent from patient-to-patient, despite considerable heterogeneity (in terms of age, gender, ethnicity, and underlying medical conditions) in the XFS/XFG patient group (Supplementary Table S1).

### Mass Spectrometric Analysis of Lens Capsule Samples

Other investigators have directly analyzed “neat” samples of XFM, dissected from the surface of the capsule.^17^ In the present study, the pieces of lens capsule were generally not heavily laden with XFM and we were unable to peel reproducibly the aggregated material from the capsule surface. Consequently, we chose to utilize intact (albeit decellularized) capsule specimens, with the goal of identifying XFM components through a comparative analysis of XFS/XFG and control samples.

In independent analyses, we compared XFS/XFG samples with either CAT samples (Experiment 1) or CAT/GL samples (Experiment 2). Of note, the two types of control sample available for this study (CAT and CAT/GL) were similar in composition. A comparative analysis of the proteomes of CAT and CAT/GL samples revealed that only 5 of more than 500 identified proteins showed significant differences in expression between the two groups (see Supplementary Table S3). The most differentially expressed protein was αB-crystallin, an abundant soluble protein of the lens, which was identified in all control samples but was particularly abundant in the CAT group.

**Figure 2.**
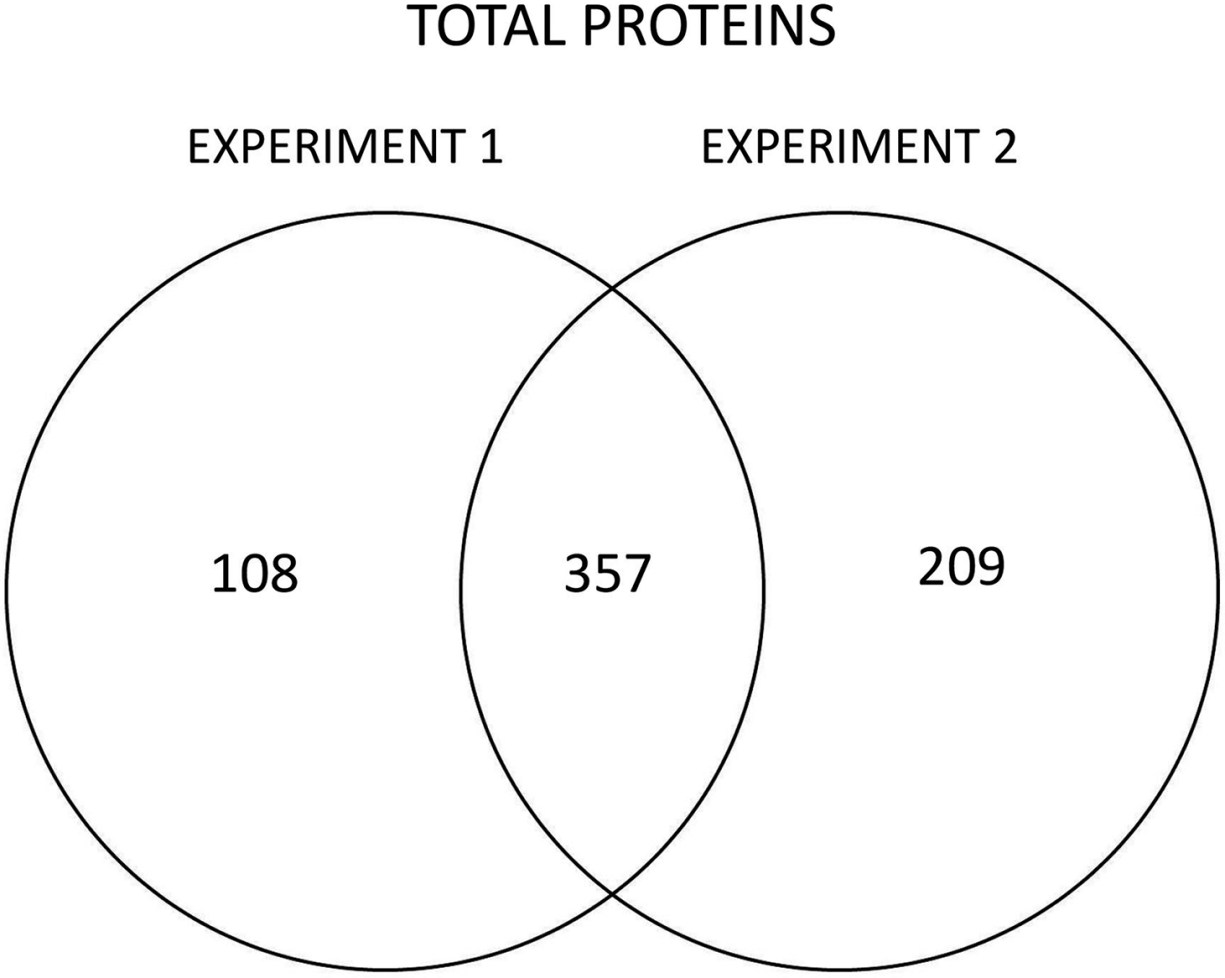
Number of proteins identified in two experiments comparing XFS/XFG and control (CAT or CAT/GL) capsular samples.

**Figure 3.**
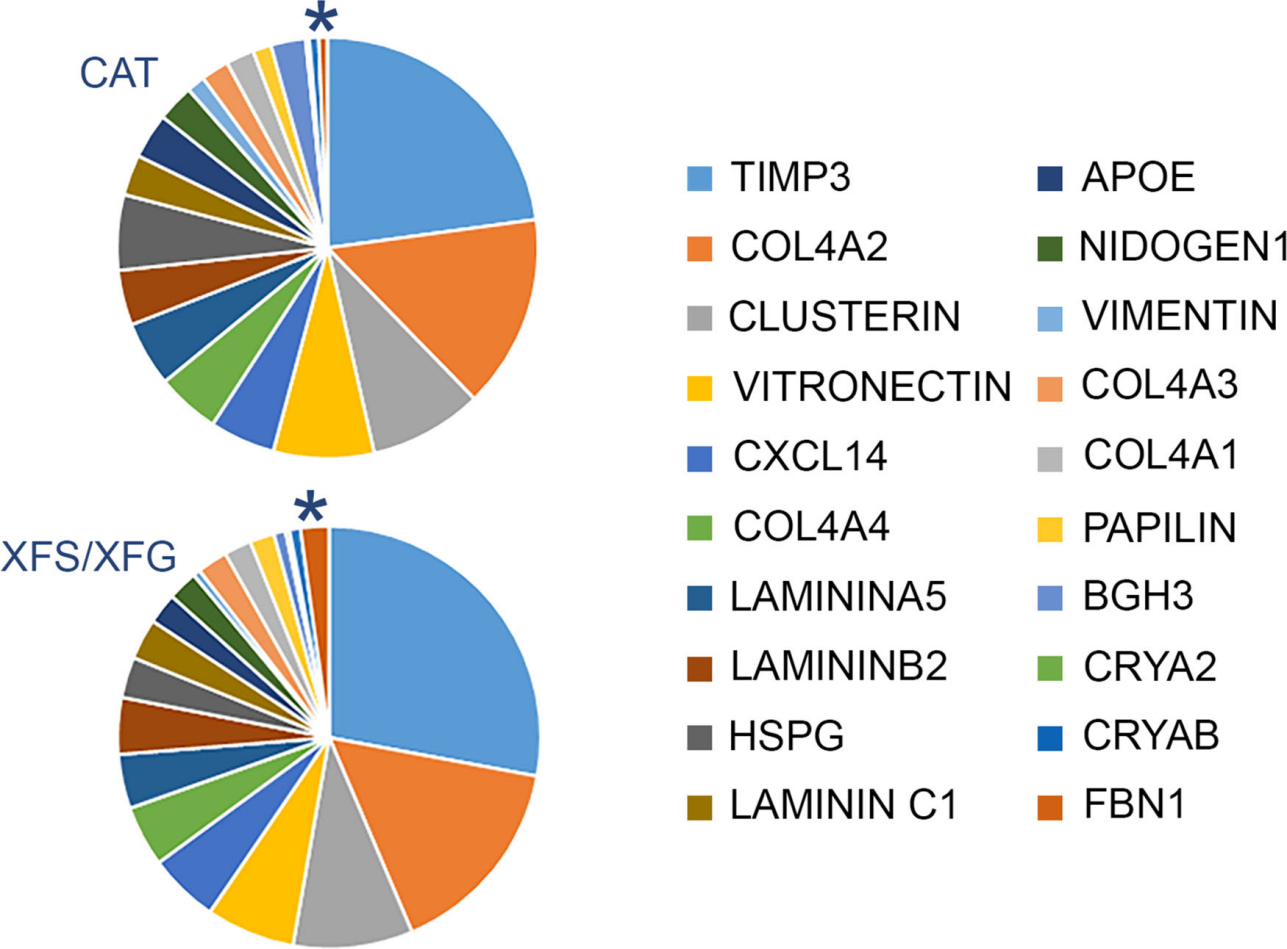
Twenty most abundant proteins in capsule specimens from cataract patients (CAT) or cataract patients with XFS or XFG. The 20 proteins account for approximately 70% of the total protein in each case. Note that the composition of the CAT and XFS/XFG samples is quite similar, although FBN1 (*) is visibly increased in the latter.

A proteomic analysis yielded 674 identified proteins in the two experiments, 357 of which were common to both (Fig. 2 and Supplementary Table S3). As expected from a sample consisting largely of lens capsule, matrisomal proteins^20^ dominated the proteome in all cases, although cytosolic proteins were also detected, albeit mostly at low levels. Among the most abundant proteins in all samples were basal lamina components such as collagen IV, laminin, basement membrane-specific heparin sulfate proteoglycan core protein, tissue inhibitor of metalloproteinases 3, clusterin, and nidogen, (Fig. 3). These proteins have been identified consistently in proteomic studies of the lens capsule.^29^ Abundant cytoplasmic lens proteins (e.g., α-crystallin and vimentin) were also commonly detected. Together, the top 20 most abundant proteins accounted for approximately 70% of the total protein. The relative proportions of these components were broadly similar in XFS/XFG and control samples (Fig. 3).

In the first experiment, we compared the proteomes of five CAT samples (#T19, T22, T23, T24, T25) and four XFS/XFG samples (#K1, GH1, S73, S74) using the TMT technique. After Benjamini–Hochberg correction for multiple comparisons, 10 proteins were identified as significantly upregulated in XFS/XFG compared with CAT samples, with medium or high confidence (*P* values of <0.05 or <0.01, respectively). The cross-linking enzyme, lysyl oxidase-like-1 (LOXL1) showed the largest (18-fold) increase in expression in XFS/XFG samples (see Table 2). Interestingly, three members of the transforming growth factor beta superfamily (TGFβ2, TGFβ3, and LEFTY2) were also upregulated in XFS/XFG samples, with LEFTY2 being particularly abundant. LEFTY2 has a role in establishing left–right body asymmetry during embryonic development,^30^ but has no known role in ocular pathophysiology. Several microfibril-associated proteins showed a significant increase in the XFS/XFG samples, including latent-transforming growth factor-binding protein-2 (LTBP-2), LTBP-3, and microfibril-associated protein-2. The expression of proteoglycan 4 (PRG4), a mucin-like glycoprotein, was increased four-fold. Fibrillin-1 (FBN1), which forms the structural backbone of microfibrils, was increased in the XFS/XFG samples but the increase did not reach significance after Benjamini-Hochberg correction. Serum amyloid-P component was one of two downregulated matrisomal proteins in the XFS/XFG capsular samples, the other being fibronectin (Supplementary Table S3).

**Table 2.** Selected Upregulated Proteins in Experiments 1 (XFS/XFG vs CAT) and 2 (XFS/XFG vs CAT/GL)^*^

Uniprot Accession |	Fold		Corrected	XFS and XFG	Control	
Protein ID	Change	*P* Value	*P* Value	(Average Intensity)	(Average Intensity)	Confidence
EXPT 1 (XFS and XFG vs CAT)						
Q08397|LOXL1	18.69	4.39E−08	2.21E−05	4.62E+06	2.47E+05	High
O00292|LFTY2	4.39	1.54E−06	3.88E−04	3.14E+06	7.17E+05	High
P10600|TGFB3	7.61	6.03E−06	9.60E−04	9.21E+04	1.21E+04	High
Q92954|PRG4	4.06	1.48E−05	1.49E−03	2.55E+05	6.29E+04	High
P61812|TGFB2	5.89	4.90E−05	4.12E−03	1.58E+05	2.68E+04	High
P25067|CO8A2	2.74	3.38E−04	2.43E−02	4.62E+04	1.68E+04	Medium
Q9NS15|LTBP3	4.43	3.99E−04	2.51E−02	1.54E+05	3.47E+04	Medium
P55001|MFAP2	3.33	4.72E−04	2.64E−02	4.38E+05	1.32E+05	Medium
Q14767|LTBP2	2.89	6.11E−04	3.08E−02	6.29E+05	2.18E+05	Medium
EXPT 2 (XFS and XFG vs CAT/GL)						
Q9H3Y0|CRSPL	18.74	2.61E−09	1.47E−06	6.33E+04	3.38E+03	High
P10600|TGFB3	13.74	1.93E−07	5.41E−05	3.17E+04	2.31E+03	High
P35555|FBN1	16.15	6.67E−07	1.30E−04	4.75E+06	2.94E+05	High
O00292|LFTY2	7.65	4.21E−06	4.7E−04	9.82E+05	1.28E+05	High
Q08397|LOXL1	13.82	1.50E−05	1.06E−03	3.19E+06	2.31E+05	High
Q14767|LTBP2	3.42	3.80E−05	2.08E−03	4.87E+05	1.42E+05	High
Q9NS15|LTBP3	3.73	3.71E−04	6.72E−03	1.53E+05	4.10E+04	High
P61812|TGFB2	4.05	4.28E−04	7.08E−03	7.31E+04	1.80E+04	High

* High confidence indicates a corrected p-value ≤ 0.01. Medium confidence indicates ≤0.05.

In the second experiment, XFS/XFG samples (#SI3, T1, K1, and S73) were compared with CAT/GL samples (#S78, S79, S80, and S83). In this case, a greater number of proteins (>50) showed statistically significant changes in expression between the XFS/XFG and control samples. A subset of these is shown in Table 2 and the complete listing is provided in Supplementary Table S3. Most of the upregulated proteins identified in the first experiment were also significantly upregulated in the second. Of note, FBN1 showed a statistically significant, sixteen-fold increase in the XFS/XFG samples in the second experiment. Several proteins in XFS/XFG samples were identified as down regulated in the second experiment. Most of these were minor cytosolic components that exhibited relatively modest (<2-fold) changes in abundance. No protein showed consistent decreases across both experiments.

**Figure 4.**
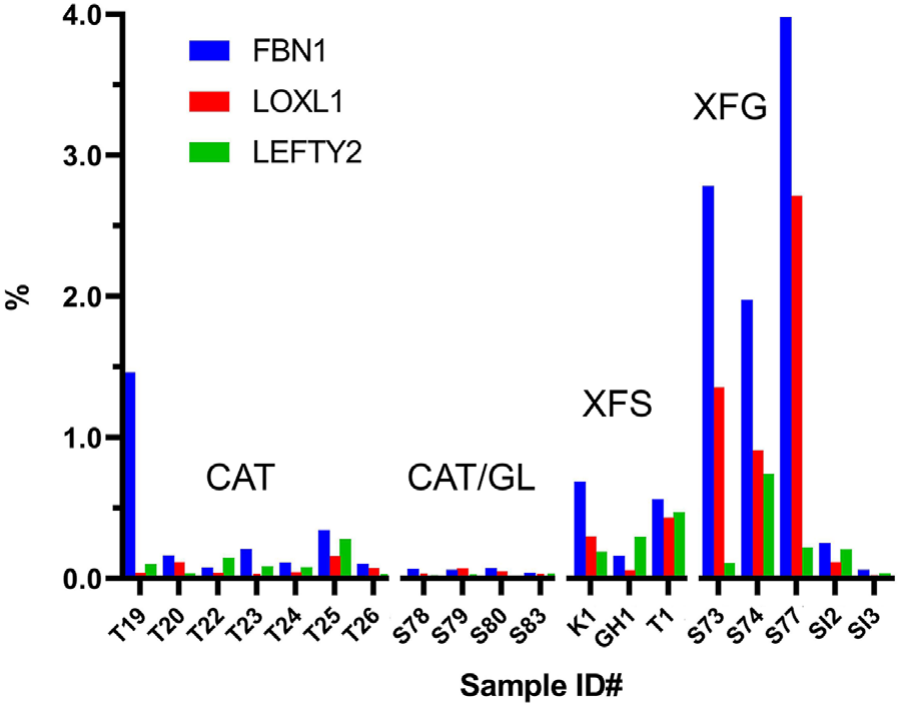
Relative abundance of three putative XFM components (FBN1, lysyl oxidase-like 1 (LOXL1), and LEFTY2, in human capsule specimens. Specimens were obtained from cataract patients (CAT), cataract patients with glaucoma (CAT/GL), cataract patients with XFS, and cataract patients with XFG. *Bars* represent the fraction of total protein present, as estimated from reporter ion intensities for that protein in each sample relative to the total reporter ion intensity for all proteins.

To determine whether the composition of XFM varied between individuals, we computed the proportional ion intensity of the differentially expressed proteins shown in Table 2. This normalization step allowed for comparisons across the two experiments. Proteins such as FBN1, LOXL1, and LEFTY2, which were barely detectable in the CAT or CAT/GL samples, were prominent in most XFS/XFG samples, where they constituted between 0.5% and 5.0% of the total protein (Fig. 4). Interestingly, samples from patients with XFS who had not (yet) developed glaucoma had noticeably less FBN1, LOXL1, and LEFTY2 than samples from patients with XFG.

**Figure 5.**
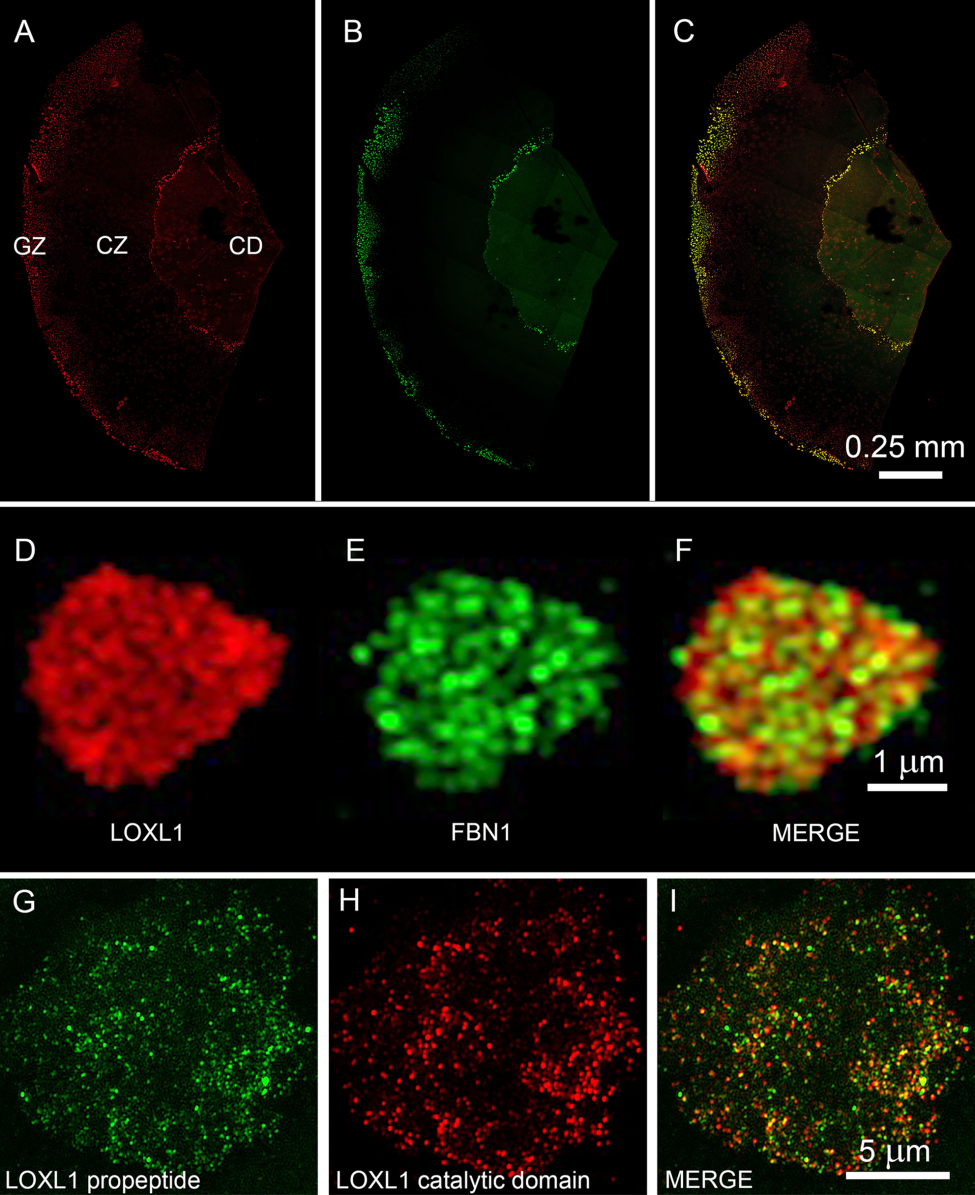
Immunofluorescence localization of LOXL1 and FBN1 on the surface of a lens capsule specimen from a patient with XFS (#K28). (**A**–**C**) Both LOXL1 (*red*) and FBN1 (*green*) label XFM in the central disc (CD) and granular zone (GZ). The clear zone (CZ), which lacks XFM, is unlabeled. The two immunofluorescence signals largely overlap, as shown in the merged image (**C**). However, high magnification images (**D**–**F**) of an individual XFM aggregate from the GZ shows that the two proteins are not precisely colocalized. Similarly, antibodies to the *N*-terminal (**G**; *green*) or *C*-terminal (**H**; *red*) regions of LOXL1 label XFM aggregates but the two signals only partially overlap (**I**).

**Figure 6.**
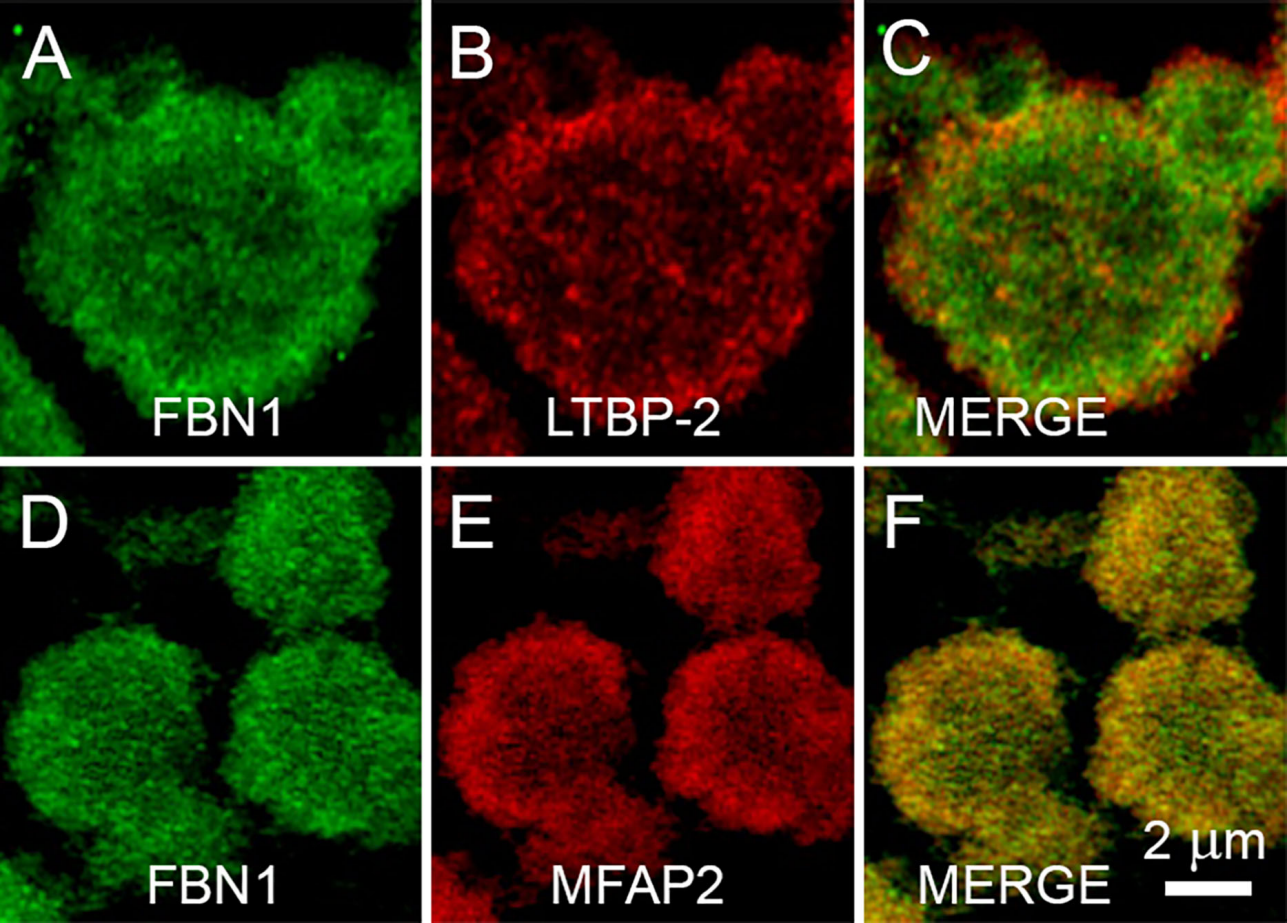
Localization of FBN1, LTBP-2 and (MFAP2) in aggregates of exfoliation material on the surface of a lens capsules from an XFG patient (#S48).

To confirm that upregulated proteins identified by mass spectrometry were bona fide XFM components, selected proteins were localized on the surface of XFS/XFG or control capsules using confocal immunofluorescence or super-resolution imaging (Figs. 5 and 6). Antibodies to FBN1 and LOXL1 labeled XFM deposits in the CD and GZ areas of XFS/XFG lens capsules. The two proteins broadly colocalized in XFM-rich regions (Figs. 5A–C), although super-resolution images (Figs. 5D–F) suggested that FBN1 and LOXL1 were only partially colocalized within individual XFM aggregates.

The mature LOXL1 enzyme becomes catalytically active following the removal of an *N*-terminal propeptide region.^31^ The propeptide is believed to facilitate the targeting of LOXL1 to its substrate.^32^ It is of interest, therefore, to determine whether the LOXL1 protein identified in the XFM deposits retained its propeptide. To visualize the fate of the propeptide and catalytic domains independently, we used antibodies raised against epitopes located in the *N*-terminal and *C*-terminal regions, respectively, of the protein. XFM aggregates were strongly labeled by both antibodies but there was relatively little overlap between the two immunofluorescence signals, implying that much of the LOXL1 is cleaved and that both domains are retained within the aggregates, albeit in separate locations (Fig. 5G–I).

Immunofluorescence labeling was also used to localize LTBP-2 and microfibril-associated protein-2, two other differentially expressed proteins identified by mass spectrometry (Table 2). Both proteins were detected in XFM aggregates, where they colocalized with FBN1 (Fig. 6). Control samples (CAT or CAT/GL) showed no significant immunofluorescence labeling with any of the antibodies to putative XFM components, and XFS specimens incubated with irrelevant antibodies were not labeled (data not shown).

**Figure 7.**
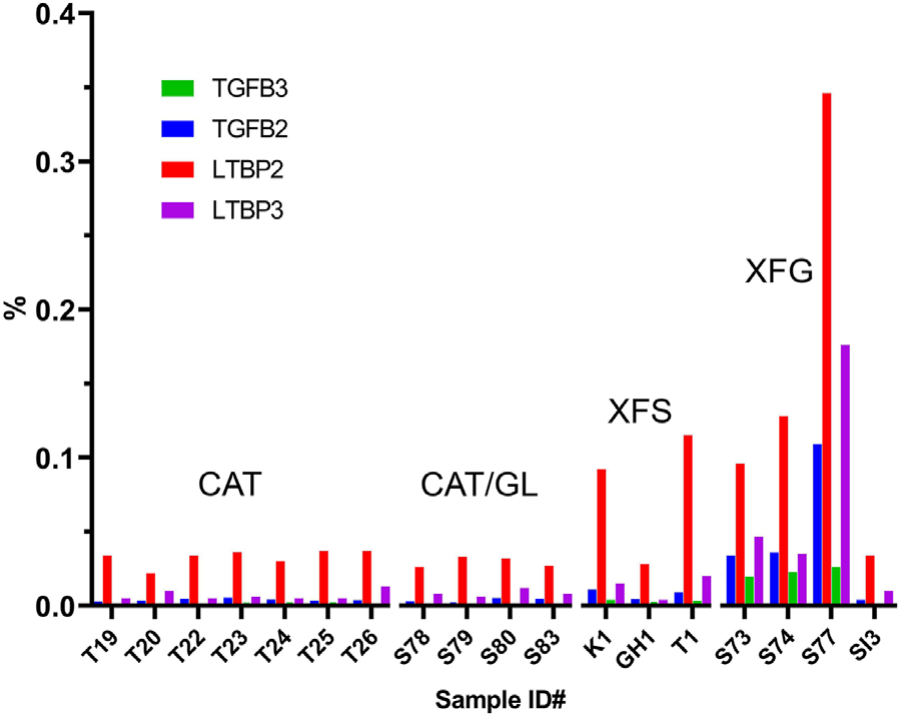
Relative abundance of TGFβ isoforms and latent TGFβ-binding proteins (LTBPs) in capsule samples from control patients (CAT and CAT/GL), a patient with XFS, and patients with XFG. Bars represent the fraction of total protein present, as estimated from reporter ion intensities for that protein in each sample relative to the total reporter ion intensity for all proteins.

LOXL1, FBN1, and LEFTY2 were the most abundant of the upregulated proteins in XFS/XFG samples but the expression of several other important matrisomal proteins was also increased. For example, TGFβ2 and TGFβ3 and two latent TGFβ-binding proteins (LTBP-2 and LTBP-3) were also identified in the XFM. The relative abundance of these proteins in individual samples is shown graphically in Figure 7.

### Mass Spectrometric Analysis of AH Samples

**Figure 8.**
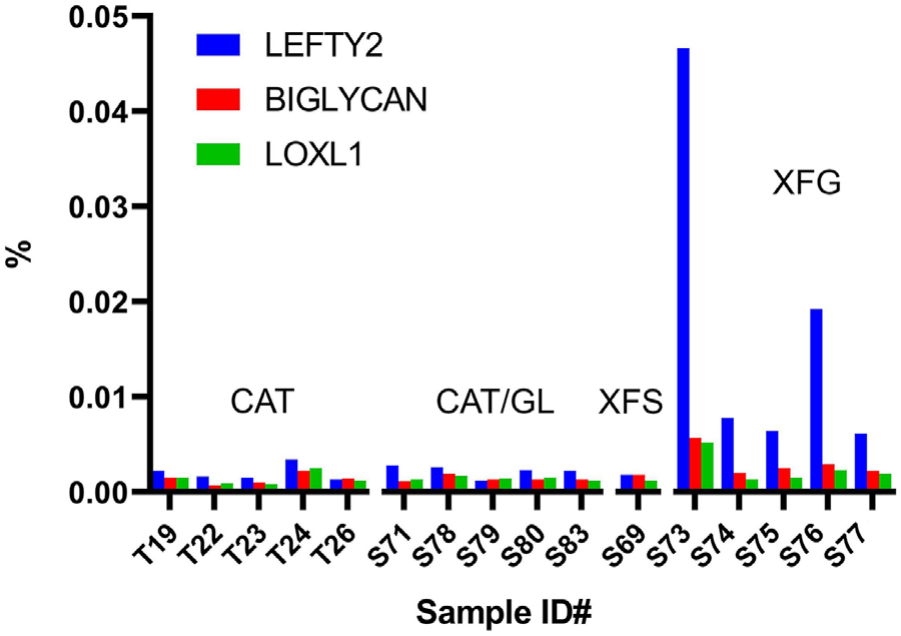
Relative abundance of LEFTY2, BIGLYCAN, and LOXL1 in AH samples from control patients (CAT and CAT/GL), a patient with XFS, and patients with XFG.

We performed a comparative analysis of AH composition in CAT, CAT/GL, patients with XFS, and patients with XFG using a single 16-plex TMTpro-based analysis. In total, 1136 different proteins were detected in AH samples (Supplementary Table S4), almost twice the number identified in the capsule samples. Consistent with previous published studies of the AH proteome,^33^^,^^34^ the most abundant components included albumin, serotransferrin, complement 3, and alpha-1-antitrypsin. Aqueous humor (AH) samples from patients with XFS and patients with XFG were compared separately to samples from CAT and CAT/GL patients. Only two proteins were identified with high confidence as being upregulated in XFS and XFG samples: LEFTY2 and PGS1 (biglycan), which on average, were increased six-fold and two-fold, respectively (Table 3). Several downregulated proteins were identified, of which one (LV743, an immunoglobulin) was significantly downregulated in comparisons of patients with XFS to patients with CAT or patients with CAT/GL. Relative expression of LEFTY2, biglycan and LOXL1 in the various AH samples is shown in Figure 8. The elevated levels of LEFTY2 and biglycan in the XFG samples compared with GAT/GL samples did not appear to reflect variations in disease severity or patient age. There was no significant difference in age, IOP, cup/disk ratio, nerve fiber layer thickness, or degree of visual field loss between the five XFG samples and the five CAT/GL samples. In each case, samples from three males and two females were included in the analysis. There was a racial difference between the samples: the patients with XFG were exclusively White, whereas three of five patients with CAT/GL were Black.

**Table 3.** Differentially Expressed Proteins in AH Samples from CAT, CAT/GL, and Patients with XFS/XFG^*^

Uniprot Accession |	Fold		Corrected	XFS	Control	
Protein ID	Change	*P* Value	*P* Value	(Average Intensity)	(Average Intensity)	Confidence
XFS and XFG VS CAT						
P21810|PGS1	2.11	3.96E−07	2.16E−4	6.37E+04	3.02E+04	High
O00292|LFTY2	6.48	2.73E−07	2.16E−04	2.94E+05	4.54E+04	High
O60814|H2B1K	−3.34	4.18E−05	7.87E−03	2.30E+04	7.70E+04	High
P68431|H31	−4.49	4.54E−05	7.87E−03	2.39E+04	1.08E+05	High
P00915|CAH1	−12.04	4.85E−05	7.87E−03	3.11E+04	3.75E+05	High
P04211|LV743	−3.15	5.70E−07	2.43E−02	9.00E+03	2.84E+04	High
P01743|HV146	−1.82	4.19E−05	7.87E−03	9.41E+03	1.71E+04	High
XFS and XFG VS CAT/GL						
P21810|PGS1	2.29	1.69E−08	9.58E−06	6.37E+04	2.77E+04	High
O00292|LFTY2	6.32	3.78E−07	1.43E−04	2.95E+05	4.66E+04	High
P04211|LV743	−4.09	1.11E−09	1.26E−06	9.00E+03	3.69E+04	High

* High confidence indicates a corrected P value of ≤0.01.

LOXL1, a major component of the XFM aggregates on the capsular surface, was only marginally increased in AH samples from patients with XFG. As with the capsular samples, increased expression of LEFTY2 and biglycan was most evident in the eyes of patients with XFS who had developed XFG.

**Figure 9.**
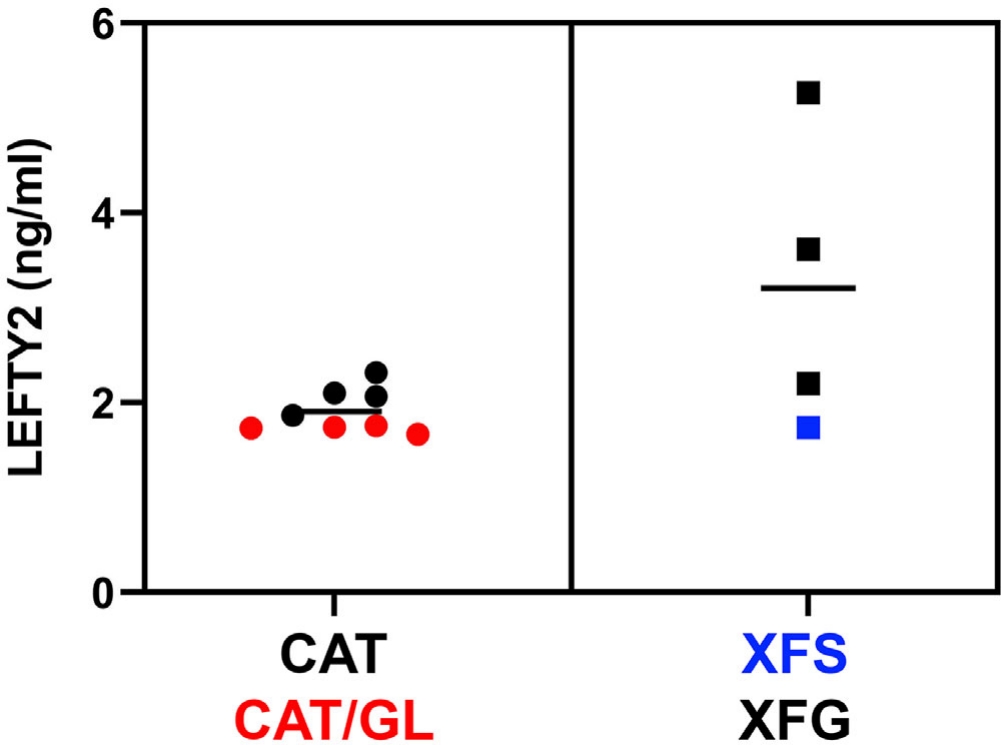
Concentration of LEFTY2 in AH samples from control (CAT, CAT/GL) or affected patients (XFS, XFG), as determined by ELISA. Horizontal line indicates the mean value in each case.

A sandwich ELISA was used to quantify the levels of LEFTY2 in the AH of patients with CAT, patients with CAT/GL, patients with XFS, and patients with XFG (Fig. 9). The concentration of LEFTY2 in the AH of affected (XFS and XFG) patients (3.21 ± 1.59 ng/mL; *n* = 4) was significantly (*P* < 0.05) elevated compared with control (CAT and CAT/GL) samples (mean, 1.91 ± 0.23 ng/mL; *n* = 8). In affected patients, the levels of LEFTY2 in the AH were highest in the patients with XFG and lowest in the single XFS patient who had not developed XFG.

## Discussion

In St. Louis, Missouri, an average of one or two patients with XFS or XFG present for cataract surgery every month to an otherwise busy university-based ophthalmology service. The prevalence of XFS in the St. Louis area thus seems to be rather low, at least compared with rates reported at higher latitudes.^35^ Nevertheless, the ophthalmologic presentation of XFS in St. Louis is indistinguishable from that described elsewhere. XFM in our samples consisted of aggregated fibrillar material deposited in a series of concentric zones on the anterior face of the lens. Ultrastructurally, XFM fibrils were about 30 nm thick, with a rough, irregular surface.

For proteomic analysis, we opted to compare capsulorhexis specimens from affected and unaffected individuals rather than to analyze “neat” samples of XFM. One advantage of our approach was that it avoided the possibility that XFM peeled from the lens surface might be contaminated with material from the superficial layers of the capsule. A potential disadvantage, however, is that bona fide XFM components that are also abundant in control capsular samples might go undetected. This factor could explain why proteins such as tissue inhibitor of metalloproteinases 3, apolipoprotein-E, and clusterin, which have been localized previously to XFM,^16^^,^^17^ were not identified here. All three were among the more abundant proteins in control capsular samples (see Fig. 3) and would have been difficult to detect in XFS/XFG specimens owing to the large background signal. Equally, however, the presence of such high levels of the three proteins in control capsules suggests that they would be likely contaminants in XFM peeled from the lens surface. In principle, this issue could be resolved using an orthogonal technique, such as immunocytochemistry, to verify the location of putative XFM proteins. This approach was taken in earlier work and used in the current study. Unfortunately, however, immunocytochemical experiments are somewhat unreliable in this setting, owing to the lack of appropriate negative controls. The use of MALDI MS imaging may provide a means to circumvent these difficulties, by allowing for spectrometric analysis of samples in situ.^36^ For the present, some caution is warranted, and the proteome of XFM should still be considered provisional.

One technical challenge in analyzing the XFM is the difficulty in solubilizing the heavily cross-linked material. Previous studies used a combination of formic acid and cyanogen bromide.^16^^–^^18^ Here, we used an alternative technique, physical disruption of the capsule by sonication in the presence of beads to shear the tissue, as well as SDS to better solubilize proteins. An S-trap digestion, which allowed for use of high concentrations of SDS in the initial protein solubilization step was also used. S-trap–based digestion has been shown to outperform traditional in-solution methods.^37^ The fact that we identified several hundred proteins in each clinical sample speaks to the general usefulness of the technique. Nevertheless, it is possible that some of the XFM remained in the insoluble fraction and as a result was not detected.

The patient group was diverse in terms of sex, age, race, and past medical history (Supplementary Table S1). Unsurprisingly, in view of this heterogeneity, protein composition varied considerably from sample to sample. Nevertheless, we were able to identify a set of proteins that were differentially expressed in patients with XFS and XFG. The two most consistently upregulated proteins were LOXL1 and FBN1. Both were detected at modest levels in control samples, where they may have been associated with the anterior tips of the zonular fibers (FBN1 and LOXL1 are known components of the ciliary zonule^38^). However, LOXL1 and FBN1 were much more abundant in the XFS/XFG samples, where their relative ion intensity values indicated that they constituted a few percent of the total protein present. Interestingly, FBN1 and LOXL1 levels were only modestly increased in patients with XFS compared with patients with XFG. Because it often takes several years for XFS to progress to XFG, it is likely that XFG samples were more heavily laden with XFM. Of note, we did not detect any proteins that were increased specifically in patients with XFS and that might have served as biomarkers for early stages of the disease.

We did not identify any proteins that were consistently downregulated in the XFS capsule samples. It has been shown previously that XFM produced by the marginal epithelial cells infiltrates the inner leaflet of the lens capsule, resulting in the formation of a so-called fibrogranular layer.^39^ Such drastic remodeling of the capsule might be expected to lead to the loss of capsular components. Although the levels of some capsule proteins (laminin, for example) were decreased in individual XFS samples, the pattern was inconsistent. This finding may suggest that marked changes in capsule composition do not accompany XFS. Alternatively, the 5-mm diameter capsule specimens may not have consistently encompassed the fibrogranular layer, which is restricted largely to the peripheral region of the capsule.^39^

In the human zonule, LOXL1 is approximately 500-fold less abundant than FBN1.^38^ In XFS samples, however, the ion intensities for the two proteins were comparable. If the relative ion intensities accurately reflect the underlying stoichiometry, then fibrillin-rich fibrils in the XFM seem to be much more heavily decorated with LOXL1 than those in the zonule. We made no attempt to account for the sizeable difference in molecular mass between LOXL1 and FBN1. Because LOXL1 is a considerably smaller protein, its relative abundance may be underestimated by the methods used here. In that case, on a molar basis, LOXL1 may be the most abundant protein in the XFM.

A recent ultrastructural analysis of negatively stained XFM revealed that the exfoliation fibrils feature a core of two or more microfibrils twisted around each other.^13^ The microfibrils have approximately the same dimensions as the fibrillin microfibrils found in the zonule and elsewhere. However, they do not seem to display the characteristic beads-on-a-string organization characteristic of negatively stained fibrillin polymers viewed by electron microscopy.^40^ If the XFM microfibrils are composed of FBN1, this finding may suggest that in the presence of high levels of LOXL1 protein, the fibrillin polymer adopts an alternative tertiary structure. Elucidating the detailed molecular organization of this novel structure, and the nature of its interaction with LOXL1, will be important in understanding the etiology of this condition.

XFM has a significant carbohydrate component, staining strongly with the periodic acid–Schiff reaction^41^ and reacting with a variety of lectins.^42^ Glycosaminoglycans such as chondroitin sulfate and dermatan sulfate are associated with the XFM deposits.^43^ Here we identified proteoglycan 4 (aka lubricin), a mucinous glycoprotein, as a significantly upregulated proteoglycan in XFS/XFG samples. proteoglycan 4 has not previously been associated with intraocular XFM deposits, although it is known to function as a boundary lubricant at the ocular surface, acting to reduce friction between the corneal surface and inner eyelid.^44^ Because proteoglycan 4 expression is upregulated in response to increased mechanical shear stress,^45^ its presence in XFM deposits could reflect increased friction between the iris pigment epithelium and the roughened surface of the XFM-coated lens capsule.

The presence of three TGFβ superfamily members (TGFβ2, TGFβ3, and LEFTY2) in XFS and XFG samples was striking. LEFTY2, a protein with a role in establishing the left–right body asymmetry during embryonic development, was expressed particularly strongly in capsules from affected patients and in AH samples. Whereas elevated levels of TGFβ2 have been reported in glaucomatous eyes,^46^ to our knowledge, LEFTY2 has not previously been associated with XFG and its presence in both the capsule and AH samples from patients with XFG was unexpected. LEFTY2 is an atypical member of the TGFβ family^47^ and serves to antagonize signaling through the nodal pathway by binding competitively to type II activin receptors.^48^^,^^49^ In addition to its well-studied role in the embryo, LEFTY2 also seems to function in the endometrium. Originally identified as an endometrial bleeding-associated factor, its transcription increases markedly during the perimenstrual phase, where it is associated with increased metalloproteinase expression.^50^ It is currently unclear whether LEFTY2 has a causal role in XFG or whether its expression is a response to the production of XFM within the eye or excavation of the optic nerve head.

The proteome of human AH has been studied extensively in healthy individuals^51^ and patients with XFS.^52^^–^^54^ Our data on samples from unaffected individuals were broadly consistent with published findings. In all samples, a few proteins (e.g., albumin and serotransferrin) dominated the proteome and most other proteins were detected at relatively low levels. Somewhat surprisingly, given the total number of detected proteins, comparison of XFS samples with CAT or CAT/GL samples identified only two consistently upregulated proteins: LEFTY2 and biglycan. LEFTY2 was also a prominent component of the XFM. In contrast, biglycan was not detected in any of the capsular samples and its cellular origin is unclear. Single-cell RNAseq analysis has shown that biglycan is expressed by cells of the human outflow pathway and, in mice, biglycan is broadly expressed in the uvea,^55^ where it localizes to choroidal blood vessels.^56^ Biglycan is usually sequestered in the extracellular matrix, but can be liberated from the matrix during stress or tissue injury, thereafter serving as part of the damage-associated molecular pattern response.^57^ Damage-associated molecular pattern signals engage the immune system, activating a plethora of downstream pathways and helping to establish a “sterile inflammation” state.^58^ Before XFM manifests in the eye it can be detected close to iridal blood vessels.^41^ It will be interesting to test whether this is the source of biglycan, or other proteins found at elevated levels in the AH. In this regard, accelerated breakdown of the blood/aqueous barrier has consistently been reported in the eyes of patients with XFS.^59^

## Conclusions

This study provided insights into the composition of the fibrillar material that accumulates on the lens surface in patients with XFS and patients with XFG. The main limitation of the study was the relatively small sample size, a reflection of the comparatively low prevalence of XFS in the local patient population. Nevertheless, we were able to confirm the identity of XFM components reported in earlier studies, while identifying novel proteins in XFM and AH samples. Some of these, including LEFTY2 and biglycan, have potential signaling roles, and could be investigated further in an effort to better understand the etiology of this condition. LEFTY2, in particular, may hold promise as a useful biomarker for XFG, if the elevated levels identified in the current work are confirmed in larger clinical studies.

## Supplementary Material

Supplement 1

Supplement 2

Supplement 3

Supplement 4
