# Supplementary material for: Compositional Analysis of Extracellular Aggregates in the Eyes of Patients With Exfoliation Syndrome and Exfoliation Glaucoma
Source: Invest Ophthalmol Vis Sci. 2021 Dec 29;62(15):27. doi: 10.1167/iovs.62.15.27 (PMC8740535; doi:10.1167/iovs.62.15.27)
Supplement: Supplement 1 [file iovs-62-15-27_s001.pdf]

Sample ID #	Gender	Age	Ethnicity	Clinical Diagnosis	Category	IOP mmHg	Cup/disk	NFL (µm)	Visual Field loss (dB)	AH proteome	Capsule proteome	AH ELISA	Confocal Microscopy	SEM
6	Male	63	Black	No Glaucoma	CAT	20	0.65						X	X
T6	Female	76	White	No Glaucoma	CAT	17	0.20						X	X
T7	Female	64	Black	Glaucoma Sus	CAT	18	0.50		-2.92					X
T8	Female	84	White	No Glaucoma	CAT	18	0.35						X	X
T9	Male	75	White	No Glaucoma	CAT	12	0.30						X	X
T10	Female	69	White	No Glaucoma	CAT	20	0.40						X	X
T11	Female	64	White	No Glaucoma	CAT	19	0.40							X
T12	Male	78	White	No Glaucoma	CAT	12	0.25							X
T13	Female	83	White	No Glaucoma	CAT	14	0.30							X
T14	Male	78	White	No Glaucoma	CAT	16	0.55							X
T19	Male	72	White	No Glaucoma	CAT	15	0.1			X	X			
T20	Female	75	White	No Glaucoma	CAT	12	0.1				X			
T21	Female	74	White	No Glaucoma	CAT	16	0.4					X		
T22	Female	71	White	No Glaucoma	CAT	18	0.2			X	X			
T23	Male	77	White	No Glaucoma	CAT	12	0.4			X	X			
T24	Male	58	White	No Glaucoma	CAT	16	0.25			X	X			
T25	Female	76	Black	No Glaucoma	CAT	12	0.50				X			
T26	Male	67	White	No Glaucoma	CAT	19	0.40			X	X			
T32	Female	81	Black	Glaucoma Sus	CAT	17	0.55	*	-3.37			X		
T36	Female	75	White	No Glaucoma	CAT	14	0.3					X		
T39	Male	85	White	No Glaucoma	CAT	15	0.4					X		
S12	Female	73	White	No Glaucoma	CAT/GL	20	0.70							X
S13	Female	70	Black	CAG	CAT/GL	14	0.65	63	-7.71					X
S28	Female	66	White	UG	CAT/GL	21	0.35	70	-9.35					X
S63	Male	66	Black	POAG	CAT/GL	18	0.8	67	-14.58			X	X	
S65	Male	71	Black	POAG	CAT/GL	13	0.95	57	-29.28			X		
S66	Male	65	Black	POAG	CAT/GL	09	0.85	61	-19.27			X		
S68	Female	66	Black	POAG	CAT/GL	22	0.55	97	-2.29			X		
S71	Female	69	Black	SOAG	CAT/GL	18	0.70	59	-23.37	X				
S78	Female	59	Black	POAG	CAT/GL	11	0.85	68	-4.40	X	X			
S79	Male	66	White	PG	CAT/GL	21	0.40	83	-2.18	X	X			
S80	Male	66	Black	POAG	CAT/GL	11	0.90	54	-27.81	X	X			
S83	Male	82	White	POAG	CAT/GL	12	0.90	*	-4.10	X	X			
K1	Female	68	Asian		XFS	15	0.65				X			
GH1	Female	78	White		XFS	14	0.45				X			
S48	Female	81	White		XFG	12	0.4						X	
S69	Female	72	White		XFS	17	0.5			X				
S85	Female	72	White		XFS	15	0.5					X		
T1	Female	83	*		XFS	16	0.6				X			
SK48	Male	82	White		XFS	12	0.4						X	X
SI1	Female	81	*	OAG	XFG	18	0.75	70	-7.14				X	X
SI2	Female	71	Black	OAG	XFG	24	0.95	58	-28.92		X			
SI3	Female	93	White	OAG	XFG	6	0.80	60	-16.35		X			
S26	Female	80	White	OAG	XFG	18	0.55	88	1.80					X
S73	Female	80	White	OAG	XFG	30	0.8	*	-0.78	X	X			
S74	Female	78	White	OAG	XFG	15	0.9	65	*	X	X			
S75	Male	74	White	OAG	XFG	15	0.9	50	-22.50	X				
S76	Male	70	White	OAG	XFG	29	0.3	89	-0.11	X				
S77	Male	71	White	OAG	XFG	15	0.5	68	-22.50	X				
S84	Male	73	Asian	CAG	XFG	10	0.85	49	-14.42			X		
S86	Female	79	White	OAG	XFG	20	0.2	78	1.87			X		
S87	Male	73	Asian	CAG	XFG	12	0.6	72	-4.23			X		

Table of participants. CAT = patients with cataract, CAT/GL = patients with cataract and glaucoma, XFS = patients with cataract and exfoliation syndrome, XFG = patients with cataract and exfoliation glaucoma, NFL=nerve fiber layer thickness, Sus = suspect, POAG = primary open angle glaucoma, CAG = angle closure glaucoma, SOAG = secondary open-angle glaucoma, UG = uveitic glaucoma, PG = pigmentary glaucoma, * data unavailable.
