# Supplementary material for: Compositional Analysis of Extracellular Aggregates in the Eyes of Patients With Exfoliation Syndrome and Exfoliation Glaucoma
Source: Invest Ophthalmol Vis Sci. 2021 Dec 29;62(15):27. doi: 10.1167/iovs.62.15.27 (PMC8740535; doi:10.1167/iovs.62.15.27)
Supplement: Supplement 2 [file iovs-62-15-27_s002.pdf]

Orbitrap Fusion settings for first 11-plex TMT experiment on capsules performed in June 2020 using instrument control software version 3.3

Document View | Tree View

Method Summary

Method Settings

Application Mode: **Peptide**
Method Duration (min): **120**

Global Parameters

Ion Source

Ion Source Type: **NSI**
Spray Voltage: **Static**
Positive Ion (V): **2000**
Negative Ion (V): **600**
Gas Mode: **Static**
Sweep Gas (Arb): **0**
Ion Transfer Tube Temp (°C): **275**
Use Ion Source Settings from Tune: **False**
FAIMS Mode: **Not Installed**

MS Global Settings

Infusion Mode: **Liquid Chromatography**
Expected LC Peak Width (s): **30**
Default Charge State: **2**
Internal Mass Calibration: **EASY-IC™**

Experiment#1 [TMT SPS-MS3]

Start Time (min): **10**
End Time (min): **115**
Cycle Time (sec): **2.5**

Master Scan:

MS OT

Detector Type: **Orbitrap**
Orbitrap Resolution: **120000**
Mass Range: **Normal**
Use Quadrupole Isolation: **True**
Scan Range (m/z): **400-1600**
RF Lens (%): **60**
AGC Target: **Standard**
Maximum Injection Time Mode: **Auto**
Microscans: **1**
Data Type: **Profile**
Polarity: **Positive**
Source Fragmentation: **Disabled**
Use EASY-IC™: **True**
Scan Description:

Filters:

Precursor Selection Range

Mass Range (m/z): **400-1600**

MIPS

Monoisotopic Peak Determination: **Peptide**

Intensity

Filter Type: **Intensity Threshold**
Intensity Threshold: **5.0e3**

Charge State

Include charge state(s): **2-6**
Include undetermined charge states: **False**

Dynamic Exclusion

Exclude after n times: **1**
Exclusion duration (s): **30**
Mass Tolerance: **ppm**
Low: **10**
High: **10**
Exclude Isotopes: **True**
Perform dependent scan on single charge state per precursor only: **True**

Data Dependent

Data Dependent Mode: **Cycle Time**
Time between Master Scans (sec): **2.5**

Scan Event Type 1:

Scan:

ddMS^2 IT CID

Isolation Mode: **Quadrupole**
Isolation Window (m/z): **2**
Isolation Offset: **Off**
Activation Type: **CID**
Collision Energy Mode: **Fixed**
CID Collision Energy (%): **35**
CID Activation Time (ms): **10**
Activation Q: **0.25**
Multistage Activation: **False**
Detector Type: **Ion Trap**
Ion Trap Scan Rate: **Rapid**
Mass Range: **Normal**
Scan Range Mode: **Auto**
AGC Target: **Standard**
Maximum Injection Time Mode: **Auto**
Microscans: **1**
Data Type: **Centroid**
Scan Description:

Filters:

Precursor Selection Range

Selection Range Mode: **Mass Range**
Mass Range (m/z): **400-1600**

Isobaric Tag Loss Exclusion

Reagent: **TMT**

Precursor Ion Exclusion

Exclusion mass width: **m/z**
Low: **50**
High: **5**

Data Dependent

Data Dependent Mode: **Scans Per Outcome**

Scan Event Type 1:

Scan:

ddMS^3 OT HCD

MS^n Level: **3**
Synchronous Precursor Selection: **True**
Number of SPS Precursors: **10**
MS Isolation Window (m/z): **2**
MS2 Isolation Window (m/z): **2**
Isolation Offset: **Off**
Activation Type: **HCD**
Collision Energy Mode: **Fixed**
HCD Collision Energy (%): **65**
Detector Type: **Orbitrap**
Orbitrap Resolution: **60000**
Mass Range: **Normal**
Scan Range Mode: **Define m/z range**
Scan Range (m/z): **100-500**
AGC Target: **Standard**
Maximum Injection Time Mode: **Custom**
Maximum Injection Time (ms): **120**
Microscans: **1**
Data Type: **Centroid**
Use EASY-IC™: **False**
Scan Description:
Number of Dependent Scans: **10**

Orbitrap Fusion settings for second 11-plex TMT experiment on capsules performed in Nov 2020 using instrument control software version 3.4

Document View | Tree View

Method Summary

Method Settings

Application Mode: **Peptide**
Method Duration (min): **120**

Global Parameters

Ion Source

Ion Source Type: **NSI**
Spray Voltage: **Static**
Positive Ion (V): **2400**
Negative Ion (V): **600**
Gas Mode: **Static**
Sweep Gas (Arb): **0**
Ion Transfer Tube Temp (°C): **305**
Use Ion Source Settings from Tune: **False**
FAIMS Mode: **Not Installed**

MS Global Settings

Infusion Mode: **Liquid Chromatography**
Expected LC Peak Width (s): **30**
Default Charge State: **2**
Internal Mass Calibration: **RunStart EASY-IC™**

Experiment#1 [TMT SPS-MS3]

Start Time (min): **10**
End Time (min): **115**
Cycle Time (sec): **2.5**

Master Scan:

MS OT

Detector Type: **Orbitrap**
Orbitrap Resolution: **120000**
Mass Range: **Normal**
Use Quadrupole Isolation: **True**
Scan Range (m/z): **400-1600**
RF Lens (%): **60**
AGC Target: **Standard**
Maximum Injection Time Mode: **Auto**
Microscans: **1**
Data Type: **Profile**
Polarity: **Positive**
Source Fragmentation: **Disabled**
Scan Description:

Filters:

Precursor Selection Range

Mass Range (m/z): **400-1600**

MIPS

Monoisotopic Peak Determination: **Peptide**

Intensity

Filter Type: **Intensity Threshold**
Intensity Threshold: **5.0e3**

Charge State

Include charge state(s): **2-6**
Include undetermined charge states: **False**

Dynamic Exclusion

Exclude after n times: **1**
Exclusion duration (s): **30**
Mass Tolerance: **ppm**
Low: **10**
High: **10**
Exclude Isotopes: **True**
Perform dependent scan on single charge state per precursor only: **True**

Data Dependent

Data Dependent Mode: **Cycle Time**
Time between Master Scans (sec): **2.5**

Scan Event Type 1:

Scan:

ddMS^2 IT CID

Isolation Mode: **Quadrupole**
Isolation Window (m/z): **2**
Isolation Offset: **Off**
Activation Type: **CID**
Collision Energy Mode: **Fixed**
CID Collision Energy (%): **35**
CID Activation Time (ms): **10**
Activation Q: **0.25**
Multistage Activation: **False**
Detector Type: **Ion Trap**
Ion Trap Scan Rate: **Rapid**
Mass Range: **Normal**
Scan Range Mode: **Auto**
AGC Target: **Standard**
Maximum Injection Time Mode: **Auto**
Microscans: **1**
Data Type: **Centroid**
Scan Description:

Filters:

Precursor Selection Range

Selection Range Mode: **Mass Range**
Mass Range (m/z): **400-1600**

Isobaric Tag Loss Exclusion

Reagent: **TMT**

Precursor Ion Exclusion

Exclusion mass width: **m/z**
Low: **50**
High: **5**

Data Dependent

Data Dependent Mode: **Scans Per Outcome**

Scan Event Type 1:

Scan:

ddMS^3 OT HCD

MS^n Level: **3**
Synchronous Precursor Selection: **True**
Number of SPS Precursors: **10**
MS Isolation Window (m/z): **2**
MS2 Isolation Window (m/z): **2**
Isolation Offset: **Off**
Activation Type: **HCD**
Collision Energy Mode: **Fixed**
HCD Collision Energy (%): **65**
Detector Type: **Orbitrap**
Orbitrap Resolution: **60000**
Mass Range: **Normal**
Scan Range Mode: **Define m/z range**
Scan Range (m/z): **100-500**
AGC Target: **Standard**
Maximum Injection Time Mode: **Custom**
Maximum Injection Time (ms): **120**
Microscans: **1**
Data Type: **Centroid**
Scan Description:
Number of Dependent Scans: **10**

Orbitrap Fusion settings for 16-plex TMT experiment on aqueous humor performed in Nov 2020 using instrument control software version 3.4

Document View | Tree View

Method Summary

Method Settings

Application Mode: **Peptide**
Method Duration (min): **140**

Global Parameters

Ion Source

Ion Source Type: **NSI**
Spray Voltage: **Static**
Positive Ion (V): **2400**
Negative Ion (V): **600**
Gas Mode: **Static**
Sweep Gas (Arb): **0**
Ion Transfer Tube Temp (°C): **305**
Use Ion Source Settings from Tune: **False**
FAIMS Mode: **Not Installed**

MS Global Settings

Infusion Mode: **Liquid Chromatography**
Expected LC Peak Width (s): **30**
Default Charge State: **2**
Internal Mass Calibration: **RunStart EASY-IC™**

Experiment#1 [TMT SPS-MS3]

Start Time (min): **10**
End Time (min): **140**
Cycle Time (sec): **3**

Master Scan:

MS OT

Detector Type: **Orbitrap**
Orbitrap Resolution: **120000**
Mass Range: **Normal**
Use Quadrupole Isolation: **True**
Scan Range (m/z): **400-1600**
RF Lens (%): **60**
AGC Target: **Standard**
Maximum Injection Time Mode: **Auto**
Microscans: **1**
Data Type: **Profile**
Polarity: **Positive**
Source Fragmentation: **Disabled**
Scan Description:

Filters:

Precursor Selection Range

Mass Range (m/z): **400-1600**

MIPS

Monoisotopic Peak Determination: **Peptide**

Intensity

Filter Type: **Intensity Threshold**
Intensity Threshold: **5.0e3**

Charge State

Include charge state(s): **2-4**
Include undetermined charge states: **False**

Dynamic Exclusion

Exclude after n times: **1**
Exclusion duration (s): **60**
Mass Tolerance: **ppm**
Low: **10**
High: **10**
Exclude Isotopes: **True**
Perform dependent scan on single charge state per precursor only: **True**

Precursor Fit

Fit Threshold (%): **70**
Fit Window (m/z): **1.2**

Data Dependent

Data Dependent Mode: **Cycle Time**
Time between Master Scans (sec): **3**

Scan Event Type 1:

Scan:

ddMS^2 IT CID

Isolation Mode: **Quadrupole**
Isolation Window (m/z): **1.2**
Isolation Offset: **Off**
Activation Type: **CID**
Collision Energy Mode: **Fixed**
CID Collision Energy (%): **30**
CID Activation Time (ms): **10**
Activation Q: **0.25**
Multistage Activation: **False**
Detector Type: **Ion Trap**
Ion Trap Scan Rate: **Rapid**
Mass Range: **Normal**
Scan Range Mode: **Auto**
AGC Target: **Standard**
Maximum Injection Time Mode: **Custom**
Maximum Injection Time (ms): **50**
Microscans: **1**
Data Type: **Centroid**
Scan Description:

Filters:

Precursor Selection Range

Selection Range Mode: **Mass Range**
Mass Range (m/z): **450-1400**

Isobaric Tag Loss Exclusion

Reagent: **TMTpro**

Precursor Ion Exclusion

Exclusion mass width: **ppm**
Low: **25**
High: **25**

Data Dependent

Data Dependent Mode: **Scans Per Outcome**

Scan Event Type 1:

Scan:

ddMS^3 OT HCD

MS^n Level: **3**
Synchronous Precursor Selection: **True**
Number of SPS Precursors: **10**
MS Isolation Window (m/z): **2**
MS2 Isolation Window (m/z): **2**
Isolation Offset: **Off**
Activation Type: **HCD**
Collision Energy Mode: **Fixed**
HCD Collision Energy (%): **55**
Detector Type: **Orbitrap**
Orbitrap Resolution: **50000**
Mass Range: **Normal**
Scan Range Mode: **Define m/z range**
Scan Range (m/z): **110-500**
AGC Target: **Custom**
Normalized AGC Target (%): **250**
Maximum Injection Time Mode: **Custom**
Maximum Injection Time (ms): **120**
Microscans: **1**
Data Type: **Centroid**
Scan Description:
Number of Dependent Scans: **10**
